# Fabrication of Antioxidant Pickering Emulsion Based on Resveratrol-Grafted Zein Conjugates: Enhancing the Physical and Oxidative Stability

**DOI:** 10.3390/foods11233851

**Published:** 2022-11-29

**Authors:** Gerui Ren, Ying Zhu, Jieyu Shi, Jiacheng Liu, Ying He, Yufan Sun, Yujing Zhan, Junfei Lv, Min Huang, Hujun Xie

**Affiliations:** School of Food Science and Biotechnology, Zhejiang Gongshang University, Hangzhou 310018, China

**Keywords:** resveratrol, covalent binding, protein modification, Pickering emulsion, antioxidant, stability

## Abstract

Lipid oxidation is still a major problem complicating the development of food emulsions. In this study, an antioxidant Pickering emulsion stabilized by resveratrol-grafted zein (Z-R) conjugates and pectin (P) complex particles was prepared. The hydrophilic pectin successfully adjusted the wettability of Z-R; when the mass ratio of Z-R to P was 2:1 (Z-R/P_2:1_), the three-phase contact angle was 90.68°, and the wettability of the particles was close to neutral. Rheological analysis showed that the emulsion formed an elastic gel structure. FTIR spectra indicated that there was a hydrogen bond and electrostatic interaction between Z-R and P. The disappearance of characteristic infrared peaks of corn oil was due to a dense protective film formed on the surface of oil drops by Z-R/P_2:1_ particles, which was confirmed by confocal laser scanning microscopy. The emulsion stabilized by Z-R/P_2:1_ had excellent physical stability at a wide range of pH values (4–9), salt ion concentrations (0.04–0.15 mol·L^−1^) and storage times (0–30 days). The anti-lipid oxidation ability of the emulsion was outstanding; after storage for 14 days at room temperature, the MDA content in the emulsion was only 123.85 μmol/kg oil. In conclusion, the Z-R/P_2:1_ particles prepared in this study can effectively stabilize a Pickering emulsion and expand the usability of the method for constructing antioxidant Pickering emulsions.

## 1. Introduction

Emulsions are generally commonplace in people’s daily diet, being present in food items such as milk, dairy beverages, cream and other popular foods. Recently, Pickering emulsions have attracted great attention from researchers because of their excellent stability and low toxicity [1,2]. Solid particles are used to stabilize Pickering emulsions instead of high-molecular-weight polymers or low-molecular-weight surfactants, which can adsorb on the oil–water interface and form a space barrier to prevent oil droplet aggregation and oxidation. Inorganic particles, such as titanium dioxide and silicon dioxide, have adverse effects on health due to their tendency to destroy nutrient transporters and intestinal microbiota in cells, produce reactive oxygen species and induce inflammation [3]. Due to food safety and biodegradability requirements, the use of inorganic particles has been severely restricted in the food industry. Nanoparticles made of natural food-grade proteins, polysaccharides and their combination are increasingly used to stabilize Pickering emulsions [4], such as sorghum gliadin [5], soybean globulin [6], aminated gelatin [7], ovalbumin [8], bovine serum albumin [9], starch nanoparticles [10], chitin nanofibrils [11] and cellulose nanocrystals [12]. The adsorption of nanoparticles in conventional emulsions is usually reversible at the oil–water interface, which makes conventional emulsions unstable and easily decomposable, and cannot effectively prevent lipid oxidation [13]. Compared with conventional emulsions, Pickering emulsions can effectively prevent aggregation, coalescence and phase separation, and exhibit high stability under lipid oxidation conditions.

Zein is the main storage protein in the endosperm of maize grains with an isoelectric point between 6.2 and 6.8 [14]. According to molecular weights, zein is divided into four types: α-zein (21–25 kDa), β-zein (17–18 kDa), γ-zein (27 kDa and 18 kDa) and δ-zein (10 kDa). The hydrophobic amino acid content of zein exceeds 50%, and includes alanine, leucine and proline, which are abundant, and lysine and tryptophan, which are lacking, resulting in its low solubility in water. Zein is a food-grade protein that has been approved by the Food and Drug Administration (FDA) for oral use. Zein has good biocompatibility, a low price and the advantages of easy surface modification, size adjustment and self-assembly behavior [15]. Therefore, zein-based nanoparticles have been widely studied. Moreover, zein nanoparticles have been used in human colorectal cancer Caco-2 and HT29-MTX cell line toxicity tests, which proved the low toxicity of the particles [16]. Due to its self-assembly ability, it is easily to form zein-based nanoparticles. Nevertheless, because of the strong surface hydrophobicity of zein, zein nanoparticles cannot effectively stabilize Pickering emulsions alone [17]. Studies have shown that hydrophilic polysaccharides can be used to adjust the wettability of zein nanoparticles and enhance their emulsifying activities and prepare stable Pickering emulsions [18,19]. Pectin is a plant polysaccharide that has attracted special attention because of its unique physical properties and biological activities. In the food industry, pectin is widely used as a thickener, gelling agent, stabilizer and food fiber human health supplement, mainly in jam, jelly and frozen food [20]. Previously, there have been studies on the development of stable Pickering emulsions through the subtle interaction between zein and pectin. Zhang et al. [21] showed that the interaction of hypermethylated pectin and zein endowed the particle dispersions with an ordered interface structure and high absolute potential value, which prevented oil droplet aggregation and enhanced the storage stability of emulsions. Li et al. [22] found that pectin could reduce the hydrophobicity of zein–proanthocyanidins nanoparticles, and the zein–proanthocyanidins–pectin ternary composite was an ideal emulsifier for stabilizing oil–water emulsions.

The oxidation of lipids in food emulsions can lead to rancidity and the loss of nutrients. In the process of production, transportation and consumption, food emulsions in various food matrices generally have different salt concentrations and pH values. Therefore, it is very important to reduce the oxidation rate of the emulsion and ensure that the emulsion remains stable under a wide pH range and various salt concentrations [23]. At present, most studies look to reduce the lipid oxidation of Pickering emulsions by loading polyphenols such as curcumin [24], epigallocatechin gallate [25], gallic acid [26] and tannic acid [27] through physical interactions. In this case, the effective adsorption of polyphenols on the surface of oil droplets becomes the key to anti-lipid oxidation of emulsions [28,29]. However, because the physical interactions between polyphenols and proteins are reversible and loose, it is still a challenge to maintain the long-term adsorption of polyphenols at the oil–water interface during practical applications. There is also a covalent interaction between polyphenols and proteins, which is irreversible and solid [30]. We hypothesized the covalent modification of proteins by polyphenols is a great way to solve this problem. In our previous studies, a conjugate comprising covalently combined resveratrol and zein was fabricated using the alkaline treatment method, and the resveratrol-grafted zein conjugate had high antioxidant activity, emulsify activity and thermal stability [31]. Moreover, the hydrophobicity of zein was also improved by the modification of resveratrol.

In this study, a novel antioxidant Pickering emulsion based on resveratrol-grafted zein conjugates (Z-Rs) was fabricated. Pectin (P) was used to adjust the wettability of the modified zein, and a composite nanoparticle (Z-R/P) was prepared, characterized and used as the antioxidant Pickering emulsion stabilizer. The effects of the oil phase volume fraction and Z-R/P particle concentration on the stability of the Pickering emulsion were investigated through droplet size, visual observation, optical microscopy and rheology analysis. Furthermore, the pH, salt concentration and oxidant stability of the Pickering emulsions were estimated. The stability mechanism of the emulsion was also explored through Fourier transform infrared spectroscopy (FTIR) and laser scanning confocal microscopy (CLSM).

## 2. Materials and Methods

### 2.1. Materials

Resveratrol and pectin were purchased from Macklin Biochemical Co., Ltd., (Shanghai, China) and Sigma-Aldrich Corp. (St. Louis, MO, USA) provided zein. Corn oil was purchased from Wuhan Baixing Biological Technology Co., Ltd., (Wuhan, China). The dialysis membranes with 1000Da MWCO were obtained from Yuanye Biological Co., Ltd., (Shanghai, China). The reagents used in the experiment were of analytical grade.

### 2.2. Preparation of Zein–Resveratrol/Pectin Z-R/P Particles

#### 2.2.1. Preparation of Zein–Resveratrol (Z-R) Conjugates

Zein–resveratrol conjugates were fabricated via inducing an alkaline reaction [31]. Zein (1 g) and resveratrol (0.2 g) were dissolved in ethanol–water solution (50 mL, 70% *v*/*v*). The pH was regulated to 9.0 with 0.1 M NaOH solution. The two solutions were stirred for 2 h, then mixed and stirred under air for 24 h. Mixed solutions were dialyzed in an ultrasonic water bath 10 times until no free resveratrol existed in the system. Ethanol was removed by rotary evaporation. Samples were lyophilized by a freeze dryer (Scieniz-10N, Ningbo Xinzhi Biotechnology company, Ningbo, China) to obtain solid powder.

#### 2.2.2. Preparation of Z-R/P Particles

Z-R (1 g) was dissolved in 70% ethanol under continuous stirring at 900 rpm, mixed and stirred for 2 h. The ethanol in the solution was removed using a RE-52C rotary evaporator (Shanghai Tianheng Instrument Co. Ltd., Shanghai, China) at 45 °C to obtain the concentrated Z-R solution. Pectin of different qualities was dissolved in distilled water stirred at 900 rpm for 2 h. The concentrated Z-R solution was added drop by drop to the pectin solution (the ratio of Z-R/P was 5:1, 4:1, 2:1, 1:1, 1:2, 1:4), and stirred for 2 h at 900 rpm. Samples were lyophilized by a freeze dryer to obtain Z-R/P particles with different mass ratios.

### 2.3. Characterization of Z-R/P Particles

#### 2.3.1. Wettability

The three-phase contact angles of zein (Z), the zein–resveratrol conjugate (Z-R), pectin (P) and Z-R/P with different mass ratio particles were measured by Theta Attension (Sweden Bioolin Technology Co., Ltd., Beijing, China). Freeze-dried powders of Z-R/P were smoothly coated on the glass with double-sided adhesive. A high-precision syringe system was used to gently drop 2 μL of pure water onto the samples. After equilibrium was reached, the shape of the droplet was recorded with a camera, and the contact angle was calculated according to the Laplace–Young equation.

$$\cos\;\theta =\frac{\gamma_{\mathrm{po}\;}{-\;\gamma }_{\mathrm{pw}}}{\gamma_{\mathrm{ow}}}$$

where γ_po_, γ_pw_ and γ*_ow_* are the interface tensions of particles–oil, particles–water and oil–water, respectively.

#### 2.3.2. Size and ζ-Potential

Based on a previous method with some modifications [32], a Zetasizer (Nano ZS, Malrvern Instruments Ltd., Worcestershire, UK) was used to analyze the size distribution and ζ-potential of zein (Z), the zein–resveratrol conjugate (Z-R), zein–resveratrol/pectin (Z-R/P_2:1_) and pectin (P) particles. Samples were dissolved in distilled water and diluted before measurement at 298 K and equilibrated for 120 s. The size of the measured samples was calculated using the Stokes–Einstein equation, and ζ-potentials were obtained using the Smoluchowski model.

#### 2.3.3. Scanning Electron Microscopy (SEM)

The microstructure of the zein–resveratrol conjugate (Z-R), zein–resveratrol/pectin (Z-R/P_2:1_) and pectin (P) particles was observed using a scanning electron microscope (SEM, SU8010, Hitachi, Japan) at an accelerating voltage of 3 kV [33]. These particles were coated with a gold-like layer and placed on double-sided tape. All particles were imaged at 3000 times magnification.

### 2.4. Preparation of Z-R/P-Stabilized Pickering Emulsions

The pre-emulsion was obtained after treating the oil–water system containing Z-R/P_2:1_ particles with a high-speed blender for 10 min (12,000 rpm, Ultra-Turrax, IKA, Germany). Then, the pre-emulsion was homogenized three times at 40 MPa with a high-pressure homogenizer (JJ30L/60, Langfang Shengtong Machinery Co., Ltd., Langfang, China) to make the emulsion uniform.

### 2.5. Characterization of Z-R/P-Stabilized Pickering Emulsions

#### 2.5.1. Size, ζ-Potential and PDI

The procedure for the measurements of size distribution and ζ-potential was the same as that detailed in Section 2.3.2. The polydispersity index (PDI) of the measured samples was calculated using the Stokes–Einstein equation.

#### 2.5.2. Optical Microscopy

The microstructure of Pickering emulsions was observed using optical microscopy (LEICA DM4000B, Wetzlar, Germany). The emulsion to be tested was added to the glass slide, and then the cover slide was slowly placed on top of it. In order to clearly observe the microscopic structure of the droplet, the magnification was adjusted to 100× [34].

#### 2.5.3. Visual Appearance

We observed the appearance changes of the emulsion before and after storage for 15 days, and took photos with a camera.

#### 2.5.4. Pickering Emulsion Type

The emulsion was decanted to the oil phase and the water phase, and the dispersion of the emulsion droplet was observed to determine the kind of Z-R/P_2:1_-stabilized Pickering emulsion. An O/W-type of Pickering emulsion can be identified if the emulsion is dispersed in water and agminated in oil. Otherwise, it is a W/O-type Pickering emulsion.

#### 2.5.5. Rheological Measurements

A rheometer (MARSIII, Thermo Fisher Scientific, Karlsruhe, Germany) was used to measure the rheological properties of Z-R/P_2:1_-stabilized Pickering emulsion in the linear viscoelastic region. The modulus of elasticity (G′) and the modulus of loss (G′′) were recorded at 1% strain using parallel steel plates with a diameter of 40 mm (fixed clearance of 0.500 mm), with oscillations ranging from 0.1 to 100 rad/s [35]. The experiments were all conducted at 25 °C.

#### 2.5.6. Fourier Transform Infrared Spectroscopy (FTIR)

All spectra were measured using an infrared spectrometer (Nicolet IS5, Thermo Scientific, Karlsruhe, Germany). Tablets were prepared using the KBr method. The scanning range of the infrared scanner was set at 4000–1000 cm^−1^.

#### 2.5.7. Confocal Laser Scanning Microscopy (CLSM)

The interface structures of Z-R/P_2:1_-stabilized Pickering emulsion droplets were determined by CLSM (Leica TCS SP2, Wetzlar, Germany). Pickering emulsions were stained by mixing Nile blue (1%), a fluorescent dye that can be excited by an argon–krypton laser (488 nm) for protein staining, and Nile red (1%), a fluorescent dye that can be excited by a helium–neon laser (633 nm) for oil staining.

### 2.6. Stability of Pickering Emulsions

#### 2.6.1. pH Value Stability

NaOH (0.1 M) and HCl (0.1 M) solutions were configurated, and then used to adjust the pH of the emulsion to 4–9. The appearance of the emulsion before and after 24 h was observed, and the droplet size and ζ-potential of the emulsion were measured after 24 h.

#### 2.6.2. Ionic Strength Stability

A NaCl solution of 0–0.4 M was prepared. A total of 5 mL of emulsion and an equal amount of NaCl solution was poured into a test tube. The appearance of the emulsion before and after 24 h was observed, and the droplet size and ζ-potential of the emulsion were measured after 24 h.

#### 2.6.3. Storage Stability

The emulsion with Z, Z-R, Z-R/P_2:1_ particles as stabilizers was prepared. The oil phase volume fraction was fixed at 5% (*v*/*v*), and the stabilizer concentration was fixed at 1.5 wt%.

#### 2.6.4. Oxidation Stability

Oxidative stability was assessed by measuring the malondialdehyde (MDA) content of a Z-R or Z-R/P_2:1_ particle-stabilized Pickering emulsion stored in the dark for two weeks at 25 °C. The corn oil was used as a control.

### 2.7. Statistical Analysis

Three parallel experiments were set up, and the results are expressed as mean ± standard errors. The data were analyzed by one-way variance (SPSS 20.0 statistical software, SPSS Inc., Chicago, IL, USA). When *p* < 0.05, the data were considered statistically significant.

## 3. Results and Discussion

### 3.1. Characterization of Z-R/P Particles

#### 3.1.1. Wettability

**Figure 1:**
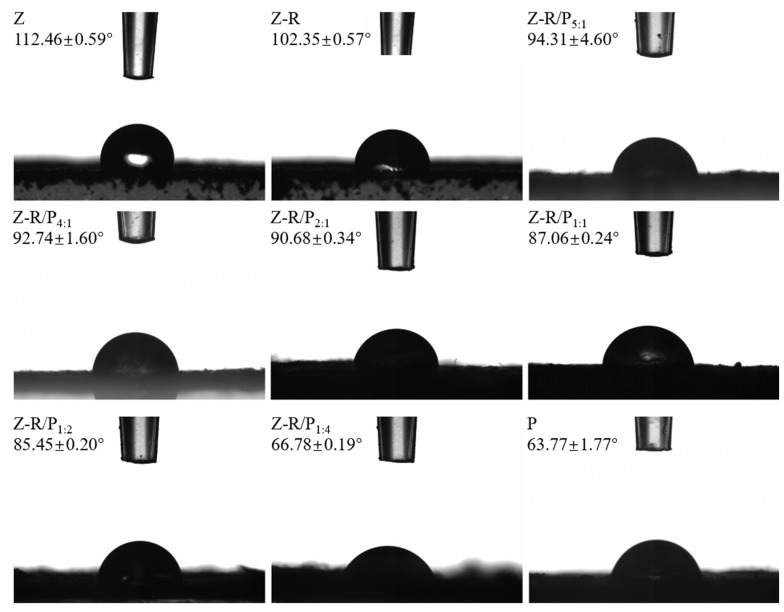
Three-phase contact angles of zein particles (Z), zein–resveratrol conjugates (Z-R), zein–resveratrol/pectin (Z-R/P) particles with different mass ratios of Z-R to P (5:1, 4:1, 2:1, 1:1, 1:2, 1:4) and pectin (P).

The three-phase contact angle is a very important parameter for the wettability of solid materials. When the three-phase contact angle is less than 90°, it indicates that the solid material has a hydrophilic surface. On the contrary, if the contact angle is more than 90°, the solid material has a hydrophobic surface. Nanoparticles with high hydrophobicity or hydrophilicity are not conducive to stabilizing Pickering emulsions, which causes most particles to be immersed in the oil phase or aqueous phase instead of adsorbed at the oil–water interface. When the contact angle of a solid particle is close to 90°, it is in a state of hydrophilic and hydrophobic equilibrium, which is suitable for the stability of the emulsion [36]. The contact angles of zein particles (Zs), zein–resveratrol conjugates (Z-Rs) and zein–resveratrol/pectin (Z-R/Ps) particles with different mass ratios of Z-R to P and pectin (P) were measured, as shown in Figure 1. Among them, the contact angle of natural zein was 112.46°, which is not much different from the research result of Zhu et al. [37], who found an angle of 113.5°, indicating that zein has high hydrophobicity and poor wettability. Compared with natural zein, the contact angle of Z-R was reduced by about 10°, which showed that the hydrophilicity of the modified zein was improved by grafting it with resveratrol. The contact angle of pectin was 63.77°, indicating it has strong hydrophilicity. For Z-R/P composite particles, the increased pectin concentration led to a gradual decrease in the contact angle. This showed that pectin plays a major role in improving the wettability of Z-R/P particles. This phenomenon was also seen in the work of Jiang et al. [38], who found that in zein nanoparticles prepared with apple pectin, apple pectin was the major contributor to wettability. When the mass ratio of Z-R to P was 2:1, the contact angle was 90.68°, which was the closest to 90°, indicating that the Z-R/P_2:1_ particle is the most favorable to stabilizing Pickering emulsions.

#### 3.1.2. Size and ζ-Potential

The mean particle size and ζ-potential of zein, Z-R conjugates, Z-R/P_2:1_ and pectin particles are shown in Table 1. The mean particle size of zein was 59.3 nm and the mean particle size of Z-R conjugates was increased to 92.6 nm. The addition of pectin significantly (*p* < 0.05) increased the particle size of the modified protein to 356.4 nm, but this was significantly (*p* < 0.05) smaller than the mean particle size of the pectin molecule (649.8 nm). This might be due to the interaction between pectin and Z-R conjugates, resulting in a more compact structure. Zhou et al. [39] reported that the size of zein/pectin particles decreased first and then increased with the increase in pectin content. This indicated that the self-assembly process of zein was affected by pectin, and pectin was not simply attached to zein. The ζ-potential value of the Z-R/P_2:1_ particle was −55.4 mV, falling between that of the Z-R conjugate and pectin. The absolute value of ζ-potential was higher than 30 mV, indicating that the dispersion stability of Z-R/P_2:1_ particles in the aqueous phase was good.

#### 3.1.3. Morphological Observation

**Figure 2:**
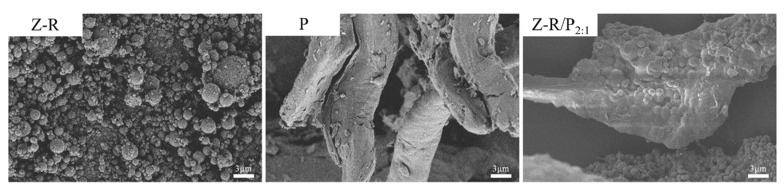
SEM images of zein–resveratrol conjugate (Z-R), zein–resveratrol/pectin (Z-R/P_2:1_) and pectin (P) particles.

The surface morphology of Z-R, Z-R/P_2:1_ and P particles was observed using a scanning electron microscope, as shown in Figure 2. Z-R particles were irregularly spherical and had many protrusions on their surface. It was speculated that the protrusions might be the result of protein self-assembly in the process of modifying zein with resveratrol. Pectin particles showed a thick and large strip structure. For the Z-R/P_2:1_ particle, the presence of pectin made the Z-R particles stick together, and the Z-R particles were wrapped into pectin. Wu et al. [40] found that nanoparticles made from Karaya gum and zein aggregate together and have irregular shapes. Polysaccharides have significant effects on the appearance and size of nanoparticle composites, and endow them with new physical and chemical properties. The adsorption of hydrophilic polysaccharide pectin on the surface of Z-R particles is thought to be beneficial to increasing the surface wettability of the Z-R particles.

### 3.2. Emulsion Formation

#### 3.2.1. Effect of Z-R/P_2:1_ Concentration on Pickering Emulsions

**Figure 3:**
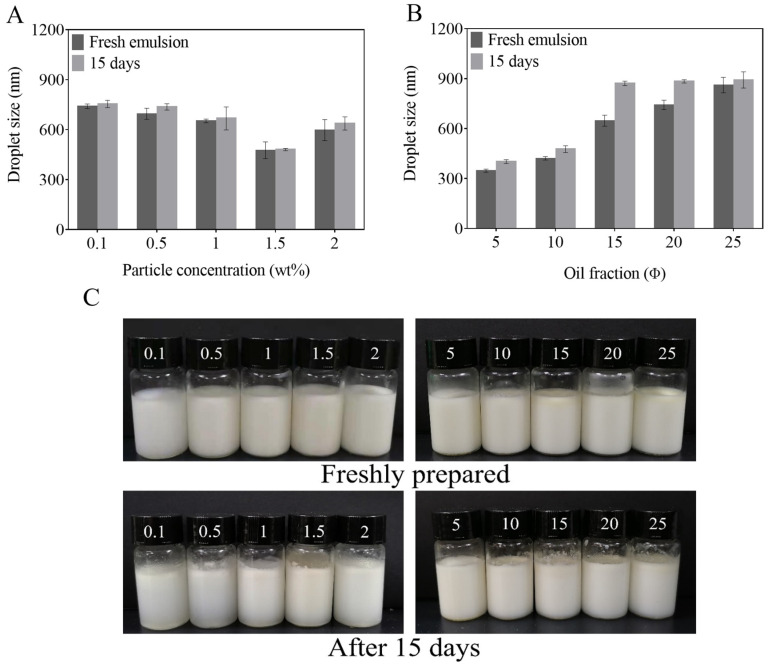
Effects of particle concentration (**A**) and oil phase fraction (**B**) on the droplet size and appearance (**C**) of Pickering emulsions stabilized by Z-R/P_2:1_ particles for 0 and 15 days of storage.

**Figure 4:**
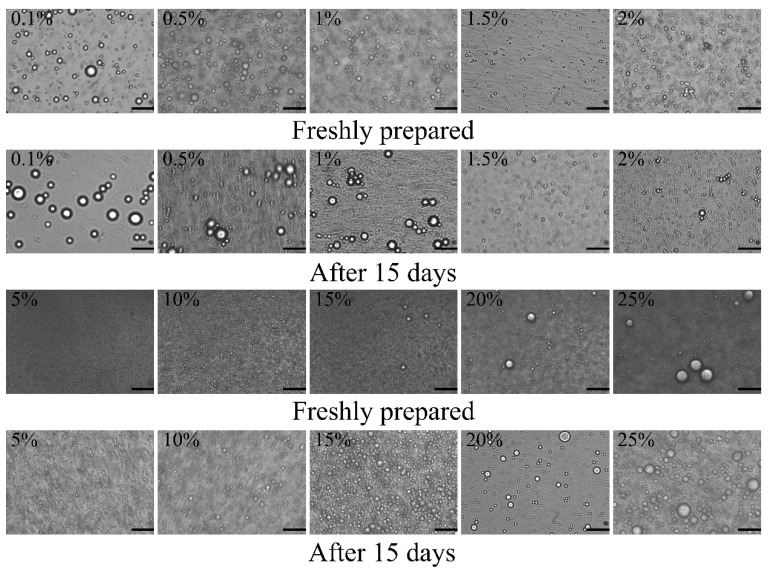
Optical microscopic images of Pickering emulsions stabilized by Z-R/P_2:1_ particles with different concentrations (0.1 wt%–2 wt%) at a fixed oil phase fraction of 5% and different oil phase volume fractions (5–25%, *v*/*v*) at a fixed Z-R/P_2:1_ particle concentration of 1.5 wt%. The scale bar is 2 μm.

The concentration of nanoparticles has a great influence on the emulsifying effect of the emulsion and appropriate particle concentration has a good inhibition effect on austenization. When the particle concentration is low, it is difficult for the emulsion droplets to form a stable interface film and the emulsion is easy to assemble and stratify due to electrostatic interaction. With increasing particle concentration, the interface area coverage of solid particles would be increased to prevent the aggregation of oil droplets [41]. The influence of the concentration of Z-R/P_2:1_ particles on the droplet size, visual appearance and microstructure of emulsions was evaluated with the volume fraction of the oil phase at ϕ = 5%. As shown in Figure 3A, the freshly prepared emulsion droplet size decreased from 740.1 nm to 476.4 nm with the increase in Z-R/P_2:1_ particle concentration from 0.1 wt% to 1.5 wt%. When the Z-R/P_2:1_ particle concentration further increased to 2 wt%, the size of emulsion droplets increased to 597.3 nm, which indicates that increasing Z-R/P_2:1_ particle concentration, up to a certain range, could prevent the condensation of droplets. However, when the concentration of stabilizer particles was too high, the micelle formed by the stabilizer could not be completely adsorbed to the surface of the droplet, which would lead to the instability of the emulsion. After 15 days of storage, there was a slight stratification phenomenon in the emulsions (Figure 3C). By comparison, the stratification of the emulsion with a Z-R/P_2:1_ particle concentration of 1.5 wt% was insignificant. Additionally, the droplet size of the emulsion with a 1.5 wt% Z-R/P_2:1_ particle concentration was basically unchanged. From the microscopic morphology of the emulsions, it could be seen that the droplets in the emulsion with a Z-R/P_2:1_ particle concentration of 1.5 wt% were small and evenly dispersed whether freshly prepared or being stored for 15 days (Figure 4). However, for other emulsions with different Z-R/P_2:1_ particle concentrations, after 15 days of storage, some of the oil droplets became larger, indicating that the phenomenon of oil droplet coalescence occurred. Therefore, the Z-R/P_2:1_ particle concentration of 1.5 wt% was selected for the subsequent study, which was conducive to increasing the stability of the emulsion in storage.

#### 3.2.2. Effect of Oil Phase Fraction on Pickering Emulsions

The volume fraction of the oil phase had a remarkable effect on the droplet size of emulsions, because it would change the number of particles that could be adsorbed on the oil–water interface. Emulsions containing different volume fractions of the oil phase (5–25%) were prepared with a fixed Z-R/P_2:1_ particle concentration of 1.5 wt%, and the effects of the volume fraction of the oil phase on the visual appearance, droplet size and microstructure of emulsions were investigated. As shown in Figure 3B, the increasing droplet size of the emulsion was affected by an increasing oil phase volume fraction. After 15 days of storage, the droplet size of the emulsion with the 5% oil phase volume fraction demonstrated little change and was still the smallest at only 401.7 nm. There was no stratification in any of the emulsions with different oil phase volume fractions; they were still uniform milky white liquids (Figure 3C). As shown in Figure 4, in the observation of optical microstructure, the increased oil phase volume fraction led to an increase in emulsion droplet size and uneven distribution, which was due to the increase in the oil phase volume fraction, and oil droplets tended to aggregate and form a lipid layer [42]. The droplet size of the emulsions with 5% and 10% volume fractions of the oil phase did not change significantly after 15 days of storage. However, in other emulsions with oil phase volume fraction of 15%, 20% and 25%, larger droplets appeared, and the aggregation phenomenon was obvious. Therefore, the volume fraction of the oil phase was selected as 5% for the follow-up study.

#### 3.2.3. Emulsion Type Judgment

The type of emulsion, O/W or W/O, was evaluated, as shown in Figure S1. The Pickering emulsion stabilized by Z-R/P_2:1_ particles was dispersed in water and gathered in oil. Hence, it was proved that this Pickering emulsion stabilized by Z-R/P_2:1_ particles was an O/W-type emulsion [43].

#### 3.2.4. Rheology Measurement

**Figure 5:**
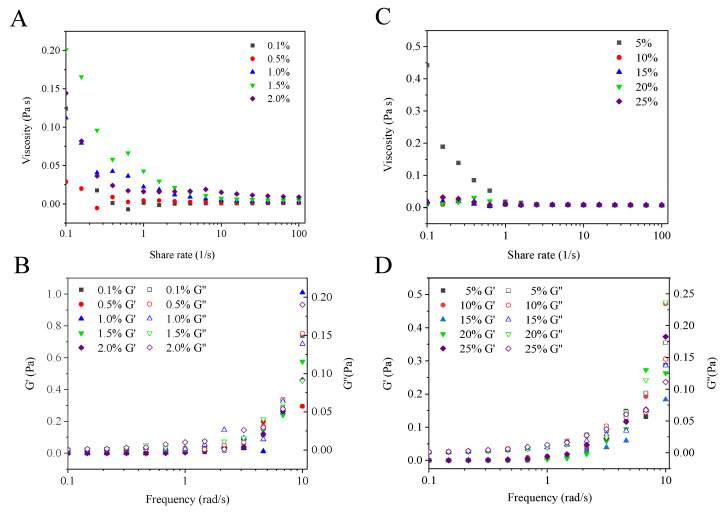
Apparent viscosity (**A**), storage modulus and loss modulus (**B**) of Pickering emulsions with different Z-R/P_2:1_ particle concentrations. Apparent viscosity (**C**), storage modulus and loss modulus (**D**) of Pickering emulsions with different oil phase volume fractions.

Rheological properties of Pickering emulsions with different Z-R/P_2:1_ concentrations and oil phase volume fractions were measured, (Figure 5). As shown in Figure 5A,C, during the increase in apparent viscosity from 0.1 to 100 s^−1^ with the shear rate, all of the emulsions exhibited shear thinning behavior. This phenomenon mainly reflected the anti-flocculation behavior of oil droplets in emulsions. The high shear rate destroyed the inner structure of droplets, and the droplets tended to reorder themselves to obtain a new equilibrium in the direction of flow [44]. In all emulsions, the storage modulus (*G*′) was higher than the loss modulus (*G*′′) in the process of increasing the frequency from 0.1 to 10 rad/s, as shown in Figure 5B,D, which indicated that the emulsion stabilized by Z-R/P_2:1_ particles had an elastic gel-like structure. 

### 3.3. Observation of Interfacial Properties

**Figure 6:**
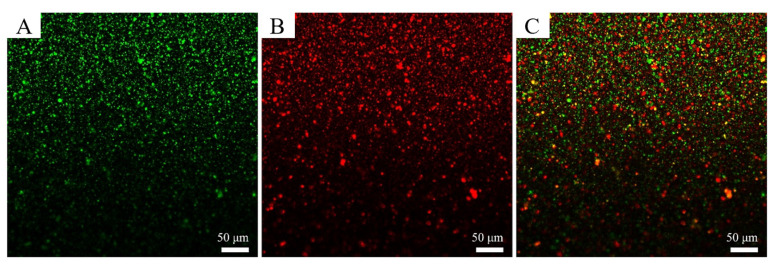
CLSM images of Z-R/P_2:1_ particle-stabilized Pickering emulsions. Oil phase dyed with Nile red (**A**), Z-R/P_2:1_ particles dyed with Nile blue (**B**), overlap image (**C**).

The interfacial microstructure of the Pickering emulsions stabilized by Z-R/P_2:1_ particles was observed by CLSM. As shown in Figure 6, Nile red was used to stain the oil phase and Nile blue was used to stain the Z-R/P_2:1_ particles. The green (Figure 6A) and red (Figure 6B) fluorescence fields represent the oil phase and Z-R/P_2:1_ particles, respectively. Figure 6C shows a superimposed fluorescence image of Pickering emulsions. The oil phase was inside the droplet, while the Z-R/P_2:1_ particles adsorbed on the border of the droplet to form a dense filling barrier which acted as a physical layer to enhance the stability of Pickering emulsions [45].

**Figure 7:**
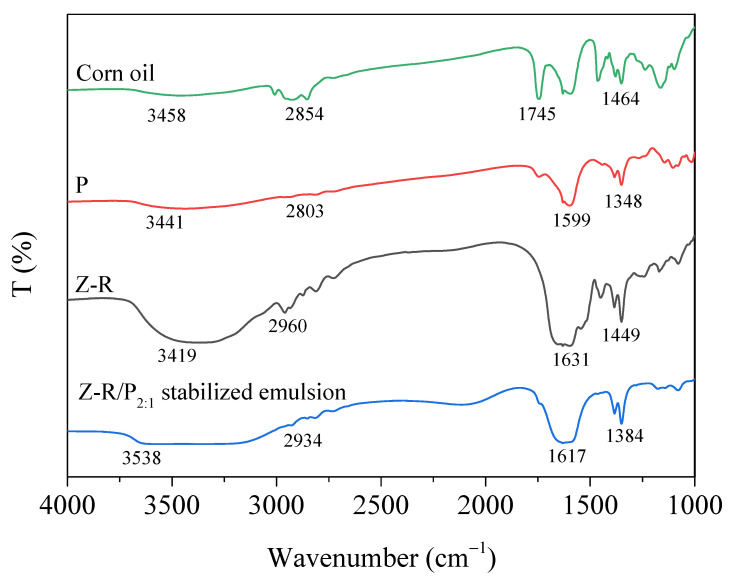
FTIR spectra of corn oil, zein–resveratrol Z-R conjugate, pectin (P) and Z-R/P_2:1_-stabilized emulsion.

FTIR is usually used to determine the interaction information between components. As shown in Figure 7, the characteristic peaks of amide I band and amide II band of Z-R were at 1631 cm^−1^ and 1449 cm^−1^, respectively, and the peak at 2960 cm^−1^ was the asymmetric stretching vibration of methylene −CH_2_ in Z-R. The three characteristic peaks of pectin were at 3441, 1599 and 1348 cm^−1^, respectively. The wide peak at 3441 cm^−1^ was the O-H stretching vibration, the 1599 cm^−1^ peak was caused by antisymmetric stretching vibration of carbonyl group in carboxylate ions, and the peak at 1348 cm^−1^ was made by the symmetric stretching vibration of the carbonyl group [46]. The three peaks of pectin changed in the Z-R/P_2:1_-stabilized emulsion, which indicated there were interactions between Z-R and pectin. The O-H peak blue shifted from 3441 cm^−1^ to 3538 cm^−1^, and the wavenumber shifted greatly, indicating that there was a strong hydrogen bond interaction between pectin and Z-R. The vibrational peaks of carboxylate ions at 1599 and 1348 cm^−1^ moved to 1617 and 1384 cm^−1^, respectively, demonstrating that there was an electrostatic interaction between pectin and Z-R. In the infrared spectrum of corn oil, the peak at 3458cm^−1^ was the stretching vibration of O-H and frequency doubling peak of C = O, while 2854 cm^−1^ was the absorption peak of methylene [47], the absorption peak at 1745 cm^−1^ was produced by the stretching vibration of carbonyl in saturated fatty acid ester, and the peak at 1464 cm^−1^ was due to the −CH_2_ bending vibration mode. However, these characteristic peaks of corn oil disappeared in the emulsion, being covered by the peaks of the Z-R/P_2:1_ complex. This might have been due to the dense protective film formed on the surface of oil drops by Z-R/P particles.

### 3.4. Determination of Emulsion Stability

#### 3.4.1. Effect of pH on Emulsion Stability

**Figure 8:**
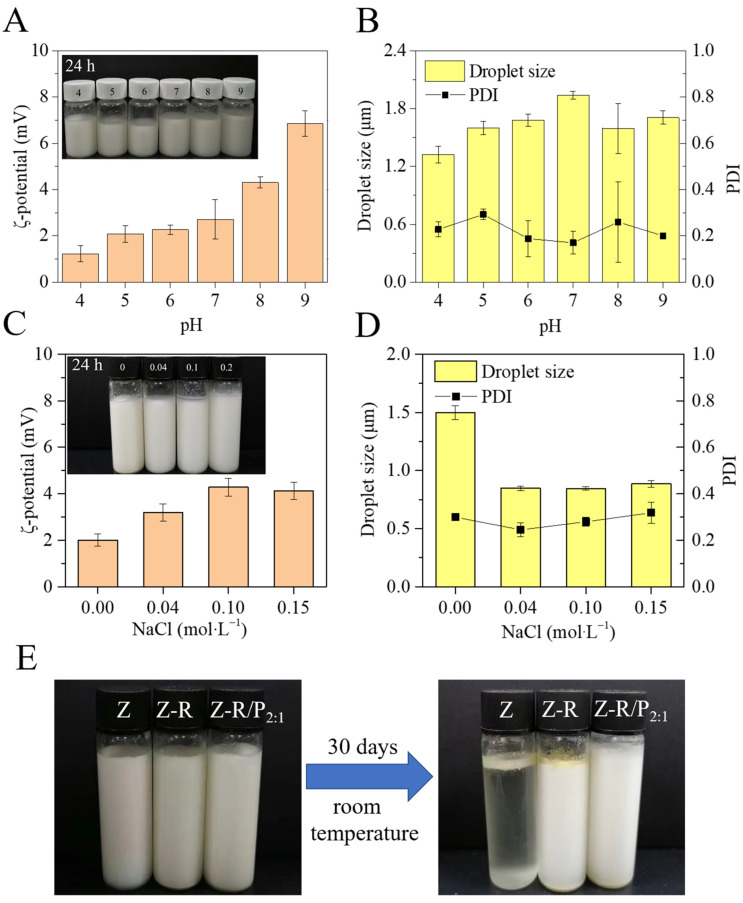
Effect of different pHs on the appearance, ζ-potential (**A**), droplet size and PDI (**B**) of Z-R/P_2:1_-stabilized emulsions after 24 h of storage. Effect of different NaCl concentrations on the appearance, ζ-potential (**C**), droplet size and PDI (**D**) of Z-R/P_2:1_-stabilized emulsions after 24 h of storage. The appearance (**E**) of emulsions stabilized by Z, Z-R and Z-R/P_2:1_ particles before and after 30 days of storage at room temperature.

The pH of food varies widely during processing, so the investigation of the effect of pH on the stability of Pickering emulsions is indispensable. The pH of the fresh emulsion was adjusted to 4–9, and the appearance, ζ-potential, droplet size and PDI of the emulsion before and after 24 h were observed (Figure 8A,B). After 24 h, the appearance of emulsions stabilized by Z-R/P_2:1_ particles at pH 4–9 was not found to change significantly and there was no stratification, which indicated that the Pickering emulsions stabilized by Z-R/P_2:1_ particles were stable at pH 4–9. The PDI values of the Pickering emulsions under different pHs were relatively small; all of them were below 0.4. The droplet size of Pickering emulsions first increased with the increase in pH values, reached the maximum at pH 7 and then decreased as the pH values increased. When the pH value was 5, being the closest value to the Z-R isoelectric point of 5.4, the emulsion still exhibited high stability towards droplet aggregation. This indicated that the interactions between Z-R and pectin were strong; thus, Z-R/P_2:1_-particle-stabilized emulsions could remain stable at a wide pH range. The ζ-potential of emulsions increased from 1.23 to 6.85 as the pH increased from 4 to 9. The addition of sodium hydroxide solution increased the pH value, and the pectin could obtain more negative charges. At pH values above the isoelectric point of Z-R, electrostatic repulsion between negatively charged Z-R and pectin inhibited the flocculation of emulsion droplets, giving the emulsion a stable state [48].

#### 3.4.2. Effect of Salt Concentration on Emulsion Stability

In the practical application of the emulsion system, it was difficult to avoid the influence of salt concentration. As shown in Figure 8C, after 24 h, when NaCl concentration reached 0.04 M, the emulsion had no stratification at all. When NaCl concentrations were 0.1 M and 0.2 M, the emulsions only had a little stratification. The salt ion stability of the emulsions stabilized by Z-R/P_2:1_ particles was good. Compared with the emulsion without added NaCl, the PDI values of the emulsions with different NaCl concentrations added did not change much (Figure 8D), which might indicate the emulsions had better salt resistance to NaCl. After adding different concentrations of NaCl to emulsions, the droplet size of the emulsions decreased obviously. This may be because the addition of NaCl in the emulsion reduced the electrostatic repulsion between non-adsorbed particles and adsorbed particles, thus promoting the adsorption of the stabilizer at the oil–water interface and reducing the size of droplets [49]. This might be because the addition of NaCl to the emulsions changed the oil–water density of the emulsions and affected the structure of water molecules, thus promoting the dissolution of the stabilizer and reducing the size of the droplets. In addition, when the NaCl concentration was 0–0.1 M, the ζ-potential of emulsions increased with the increase in NaCl concentration, which might be on account of the salt dissolution phenomenon. When the NaCl concentration increased to 0.2 M, the ζ-potential of the emulsion slightly decreased, which might have been caused by the electrostatic shielding effect or slight salting out as the NaCl concentration further increased, resulting in the reduction in ζ-potential.

#### 3.4.3. Effect of Storage Time on Emulsion Stability

The appearance of emulsions stabilized by different particles before and after 30 days of storage at room temperature is shown in Figure 8E. For emulsions stabilized by zein particles, the layer of emulsification almost disappeared after 30 days of storage, which proved that zein particles could not be used as a stabilizer alone to stabilize Pickering emulsions. This phenomenon was also confirmed by Zhang et al. [50], who found that after 28 days of storage, the droplet size increased significantly and the soybean oil was released on the top of the Pickering emulsion due to the low surface activity of zein. However, the emulsion stabilized by Z-R particles showed slight stratification and a small number of Z-R particles accumulated on the top of the emulsion. This enhanced emulsification was attributed to the increased wettability of Z-R particles. Additionally, after 30 days of storage, the appearance of the Z-R/P_2:1_-stabilized emulsion showed no change at all, and the emulsion was still uniform with no stratification. The results show that the Pickering emulsion prepared with Z-R/P_2:1_ particles as the stabilizer had excellent storage stability.

### 3.5. Oxidation Stability

**Figure 9:**
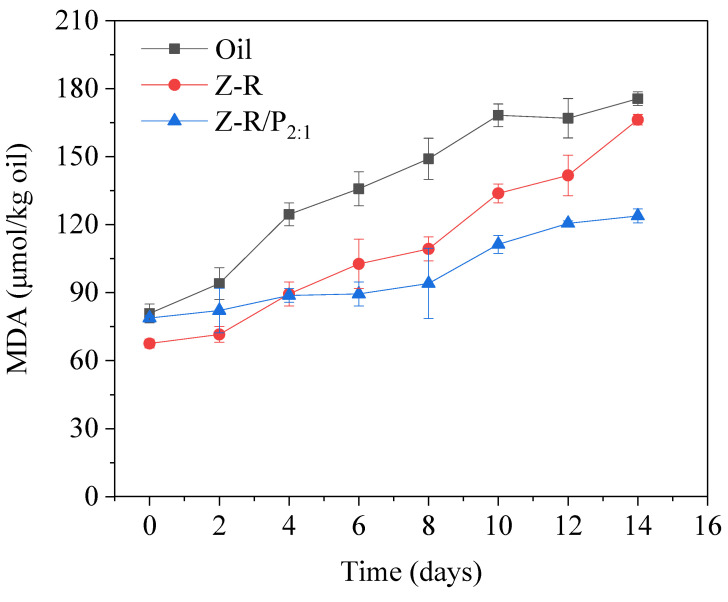
Evolution of malondialdehyde (MDA) under storage at room temperature for 14 days in Z-R-particle-stabilized Pickering emulsion and Z-R/P_2:1_-particle-stabilized Pickering emulsion, with corn oil without stabilizer used as a control.

Lipid oxidation is a major challenge in maintaining food quality during food storage and emulsion development in the food industry [51]. It will lead to degradation of bioactive substances in the oil phase, thereby reducing the quality and nutritional value of the emulsion. The oxidative stability of emulsions stabilized by Z-R and Z-R/P_2:1_ particles was evaluated by monitoring the content of malondialdehyde (MDA) during storage at room temperature, using corn oil in bulk as a control (Figure 9). As expected, the MDA contents in the emulsions stabilized by Z-R and Z-R/P_2:1_ were significantly inhibited compared with that in corn oil. Additionally, the content curve of MDA in the emulsion stabilized by Z-R/P_2:1_ was the most smooth and steady. We were concerned that during the first four days of storage, the content of MDA in the Z-R/P_2:1_-stabilized emulsion would be higher than that in the Z-R-stabilized emulsion due to the presence of pectin in the outer layer of Z-R/P_2:1_ particles of the freshly prepared emulsion, which might have hindered the role of the resveratrol molecule in antioxidation. With the extension of storage time, the interactions between pectin and Z-R gradually weakened due to the influence of environmental factors on the particles, which led to the slow release of the antioxidant functional component. After 14 days of storage, the content of MDA in corn oil had reached 175.53 μmol/kg, while that in the emulsion stabilized by Z-R slightly dropped to 166.26 μmol/kg, and that in the emulsion stabilized by Z-R/P_2:1_ was greatly reduced to 123.87 μmol/kg. It is worth noting that the content of MDA determined in this study was lower than that of conventional emulsifier-stabilized emulsions. For example, Cheng et al. [52] found that the MDA content of secoisolariciresinol nanoemulsions was about 0.25 mmol/kg after storage for 12 days. In the study by Huang et al. [53], the MDA content of a purple-sweet-potato-particle-stabilized Pickering emulsion increased by about 200 μmol/kg after 14 days of storage at room temperature. These results indicate that the Pickering emulsion stabilized by Z-R/P_2:1_ had an excellent inhibitory effect on lipid oxidation. This was due to the irreversible covalent interaction between zein and resveratrol, which enabled resveratrol to be effectively controlled at the oil–water interface of the emulsion, thus playing an outstanding antioxidant role. Liu et al. [54] also found that the covalent irreversible binding of chlorogenic acid and lactoferrin was conducive to the adsorption and accumulation of polyphenols at the emulsion interface.

## 4. Conclusions

In this study, a zein and resveratrol covalent complex was combined with pectin to form protein–polyphenol/polysaccharide composite particles (Z-R/P) to stabilize a Pickering emulsion of corn oil. Pectin could adjust the wettability of Z-R particles well, and the particles of Z-R/P_2:1_ had a three-phase contact angle of 90.68° and neutral wettability. When the concentration of Z-R/P_2:1_ particles was 1.5 wt% and the volume fraction of the oil phase was 5%, the Pickering emulsion stabilized by Z-R/P_2:1_ particles had excellent pH, salt ions and storage stability. Furthermore, the irreversible covalent interaction between zein and resveratrol could effectively control the adsorption of resveratrol at the oil–water interface of the emulsion. This was confirmed by FTIR and CLSM experiments. Most importantly, the highly efficient adsorption of resveratrol at the oil–water interface endowed the emulsion with excellent oxidation stability. This study provided a preparation method for an antioxidant Pickering emulsion stabilized by a protein–polyphenol covalent conjugate and polysaccharide. In addition, the prepared composite particles can be used as an effective new stabilizer, which further increases the application prospects of the covalent conjugate system.

## Figures and Tables

**Table 1:** The mean size distribution and ζ-potential of zein (Z), zein–resveratrol conjugate (Z-R), zein–resveratrol/pectin (Z-R/P_2:1_) and pectin (P) particles.

Samples	Size (nm)	ζ-Potential (mV)
Z	59.3 ± 6.01 ^d^	47.5 ± 3.26 ^c^
Z-R	92.6 ± 11.6 ^c^	−62.1 ± 2.77 ^a^
Z-R/P_2:1_	356.4 ± 8.32 ^b^	−55.4 ± 2.54 ^b^
P	649.8 ± 5.78 ^a^	−43.2 ± 3.45 ^c^

Values are means ± SD (n = 3). Different letters within the same column are statistically different (*p* < 0.05).

## Data Availability

The data presented in this study are available on request from the first author.

## Supplementary Materials

The following are available online at https://www.mdpi.com/article/10.3390/foods11233851/s1, Figure S1. Judgment of emulsion type.
