# Supplementary material for: Fabrication of Antioxidant Pickering Emulsion Based on Resveratrol-Grafted Zein Conjugates: Enhancing the Physical and Oxidative Stability
Source: Foods. 2022 Nov 29;11(23):3851. doi: 10.3390/foods11233851 (PMC9737855; doi:10.3390/foods11233851)
Supplement: Supplementary file 1 [file foods-11-03851-s001.zip › foods-2034078-supplementary.pdf]

Fabrication of antioxidant Pickering emulsion based on resveratrol-grafted zein conjugates: Enhancing the physical and oxidative stability

Gerui Ren, Ying Zhu, Jieyu Shi, Jiacheng Liu, Ying He, Yufan Sun, Yujing Zhan, Junfei Lv, Min Huang, Hujun Xie*

School of Food Science and Biotechnology, Zhejiang Gongshang University, Hangzhou 310018, People's Republic of China

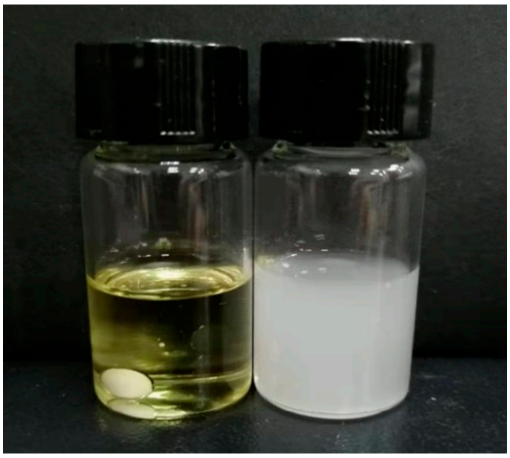

Fig. S1. Judgment of emulsion type.
